# Supplementary material for: A matter of principle or a matter of money? How fairness evaluations change with experimental currencies
Source: PLoS One. 2026 Feb 13;21(2):e0336459. doi: 10.1371/journal.pone.0336459 (PMC12904568; doi:10.1371/journal.pone.0336459)
Supplement: S2 Table — Note: All dependent variables are the evaluations after learning the monetary value of tokens, ranging from –3 to 3. (2-3) “Realized = Preferred” and “Realized ≠ Preferred” report the estimations for the subsamples in which participants’ preferred and realized redistributions were identical or not, respectively. (4-5) “Redistributed” and “Not Redistributed” report the estimation for the subsamples in which tokens of any type were redistributed or in which none of the tokens were redistributed. Controls include age, gender and features of the between-subject variation described in [33]: whether the redistribution is decided by a human, whether participants could choose the decision maker (DM), whether minimal group paradigm was used and whether the participant would face a positive, negative or no discrimination in the match based on the group affiliations. (PDF) [file pone.0336459.s002.pdf]

S2 Table. OLS regression: Adjustment of satisfaction rankings after learning the token value

	(1) Adjusted Satisfaction	(2) Adj. Satisfaction: Realized = Preferred	(3) Adj. Satisfaction: Realized $\neq$ Preferred	(4) Adj. Satisfaction: Redistributed	(5) Adj. Satisfaction Not Redistributed
Initial Satisfaction	0.6447^{***}	0.5860^{***}	0.5680^{***}	0.6210^{***}	0.6418^{***}
	(0.05)	(0.11)	(0.07)	(0.07)	(0.08)
Final Payoff (in cents)	0.0027^{***}	0.0009	0.0033^{***}	0.0027^{***}	0.0022^{***}
	(0.00)	(0.00)	(0.00)	(0.00)	(0.00)
Original Payoff (in cents)	-0.0008^{*}	-0.0005	-0.0016^{**}	-0.0010^{*}	
	(0.00)	(0.00)	(0.00)	(0.00)	
Preferred Distribution (in cents)	-0.0009^{**}			-0.0005	-0.0013^{***}
	(0.00)			(0.00)	(0.00)
Partner Payoff (in cents)	-0.0002	-0.0004	-0.0002	-0.0006	0.0001
	(0.00)	(0.00)	(0.00)	(0.00)	(0.00)
Constant	-0.3232	1.3433	-1.7363^{*}	0.0085	-0.5817
	(0.55)	(0.68)	(0.78)	(0.82)	(0.76)
Age and Gender	yes	yes	yes	yes	yes
Human DM	yes	yes	yes	yes	yes
Could choose DM	yes	yes	yes	yes	yes
Group Information	yes	yes	yes	yes	yes
Direction of Bias	yes	yes	yes	yes	yes
Adjusted R-sq	0.65	0.47	0.62	0.64	0.66
Observations	334	140	194	192	142

Standard errors clustered at the individual level in parentheses.

* $p < 0.05$, ** $p < 0.01$, *** $p < 0.001$.
