# Supplementary material for: A matter of principle or a matter of money? How fairness evaluations change with experimental currencies
Source: PLoS One. 2026 Feb 13;21(2):e0336459. doi: 10.1371/journal.pone.0336459 (PMC12904568; doi:10.1371/journal.pone.0336459)
Supplement: S1 Fig — Initial evaluations. Note: This is an example of the screens as they were presented to participants to elicit their initial evaluations of fairness and satisfaction with the redistribution. The layout is an exact representation, the text is a translation as experiment was conducted in German. Complete instructions can be found at OSF: https://osf.io/jucev/. (PDF) [file pone.0336459.s003.pdf]

Results Round 1 Decision of Player D

In Round 1, **participant D (Klee)** determined the following distribution for your group:

Players	Picture	Green Points		Yellow Points		Blue Points	
		Initial points	Redistributed points	Initial points	Redistributed points	Initial points	Redistributed points
You (Player A)	Klee	100	100	200	100	0	0
The other participant (Player B)	Klee	100	100	0	100	0	0

How satisfied are you with the decision of **participant D (Klee)**

very satisfied	satisfied	rather satisfied	neither satisfied nor dissatisfied	rather dissatisfied	dissatisfied	very dissatisfied
○	○	○	○	○	○	○

How fair do you find the decision of **participant D (Klee)**

very fair	fair	rather fair	neither fair nor unfair	rather unfair	unfair	very unfair
○	○	○	○	○	○	○

Could you explain your answer? (optional)

Next
