# Supplementary material for: A matter of principle or a matter of money? How fairness evaluations change with experimental currencies
Source: PLoS One. 2026 Feb 13;21(2):e0336459. doi: 10.1371/journal.pone.0336459 (PMC12904568; doi:10.1371/journal.pone.0336459)
Supplement: S2 Fig — Adjusted evaluations. Note: This is an example of the screens as they were presented to participants to elicit their adjusted evaluations of fairness and satisfaction with the redistribution. The layout is an exact representation, the text is a translation as experiment was conducted in German. Complete instructions can be found at OSF: https://osf.io/jucev/. (PDF) [file pone.0336459.s004.pdf]

Final Results

In Round 1, **participant D (Klee)** determined the following distribution for your group.

Players	Picture	Green Points		Yellow Points		Blue Points	
		Initial points	Redistributed points	Initial points	Redistributed points	Initial points	Redistributed points
You (Player A)	Klee	100	100	200	100	0	0
The other participant (Player B)	Klee	100	100	0	100	0	0

Given the values of the points, your earnings are as follows (in euros):

Players	Picture	Green Points	Yellow Points	Blue Points	Total
You (Player A)	Klee	4.0	2.0	0.0	6.0
The other participant (Player B)	Klee	4.0	2.0	0.0	6.0

How satisfied are you with the decision of **participant D (Klee)**

very satisfied	satisfied	rather satisfied	neither satisfied nor dissatisfied	rather dissatisfied	dissatisfied	very dissatisfied
○	○	○	○	○	○	○

How fair do you find the decision of **participant D (Klee)**

**very fair**	**fair**	**rather fair**	**neither fair nor unfair**	**rather unfair**	**unfair**	**very unfair**
○	○	○	○	○	○	○

Continue to questionnaire
