# Supplementary material for: A matter of principle or a matter of money? How fairness evaluations change with experimental currencies
Source: PLoS One. 2026 Feb 13;21(2):e0336459. doi: 10.1371/journal.pone.0336459 (PMC12904568; doi:10.1371/journal.pone.0336459)
Supplement: Attachment — Submitted filename: Response Letter PlsONe_MoM_Chug_luh.pdf [file pone.0336459.s005.pdf]

Prof. Dr. Wolfgang J. Luhan
School of Accounting Economics and Finance
Faculty of Business and Law
University of Portsmouth
Richmond Building, Portland Street
Portsmouth, Hampshire PO1 3DE|UK
+44 (0)23 9284 4129|wolfgang.luhan@port.ac.uk

Response letter: PONE-D-25-55896

A Matter of Principle or a Matter of Money? How Fairness Evaluations Change with Experimental Currencies.

Dear Dr. Dear Dr Timilsina, dear Reviewers,

thank you very much for your comments and suggestions and for infinity us to submit a revised version. We have addressed all comments and we reply to the comments and suggestions and outline the changes in detail below. The changes can be easily identified in the marked-up copy of the manuscript.

All page- and line numbers in our responses below refer to the manuscript without tracked-changes.

Format and typesetting changes:

1. Please ensure that your manuscript meets PLOS ONE's style requirements, including those for file naming. The PLOS ONE style templates can be found at

https://journals.plos.org/plosone/s/file?id=wjVg/PLOSOne_formatting_sample_main_body.pdf and

https://journals.plos.org/plosone/s/file?id=ba62/PLOSOne_formatting_sample_title_authors_affiliations.pdf

We have adjusted the manuscript to PLOS ONE's style requirements.

2. Thank you for stating the following financial disclosure:
The authors gratefully acknowledge funding by the Nuffield Foundation (FR-000000326) and DFG CRC TRR 190 (project number 280092119). Please state what role the funders took in the study. If the funders had no role, please state: "The funders had no role in study design, data collection and analysis, decision to publish, or preparation of the manuscript."
If this statement is not correct you must amend it as needed.
Please include this amended Role of Funder statement in your cover letter; we will change the online submission form on your behalf.

We have included the proposed statement in paragraph 4 of the cover letter.

3. Please expand the acronym "DFG CRC TRR" (as indicated in your financial disclosure) so that it states the name of your funders in full.
This information should be included in your cover letter; we will change the online submission form on your behalf.

We have included the full name of all funders in the funding statement in paragraph 4 of the cover letter.

4. Please include your full ethics statement in the ‘Methods’ section of your manuscript file. In your statement, please include the full name of the IRB or ethics committee who approved or waived your study, as well as whether or not you obtained informed written or verbal consent. If consent was waived for your study, please include this information in your statement as well.

We have included the ethics statement on lines 164-166. The statement about written and informed consent can be found on lines 174 to 175.

5. We noted in your submission details that a portion of your manuscript may have been presented or published elsewhere. Please clarify whether this publication was peer-reviewed and formally published. If this work was previously peer-reviewed and published, in the cover letter please provide the reason that this work does not constitute dual publication and should be included in the current manuscript.

In the response letter, we have now clarified that this manuscript is using data from an experiment of which parts were formally published in a peer-reviewed journal. In paragraph 4, we explain that while we report data from the same experimental design, none of the results or text presented here were part of this publication, and thus this is not a dual publication.

6. Please update your submission to use the PLOS LaTeX template. The template and more information on our requirements for LaTeX submissions can be found at http://journals.plos.org/plosone/s/latex.

We have updated the manuscript using the PLOS LaTeX template.

7. Please include captions for your Supporting Information files at the end of your manuscript, and update any in-text citations to match accordingly. Please see our Supporting Information guidelines for more information: http://journals.plos.org/plosone/s/supporting-information.

We have added captions and in-text citations to the Supporting Information.

Reviewer #1:

Clarify the rationale for task design (luck–effort–talent).
On page 6 (lines 138–141), the manuscript states:

“Participants could earn 0 or 100 tokens via a coin toss (luck); they counted zeros in two matrices (effort); finally, they could earn 100 tokens for solving one Raven’s Progressive Matrix (talent).”

These tasks are appropriate and intuitive, but the paper would benefit from a slightly fuller justification grounded in prior experimental literature. In particular, a short paragraph

explaining why these tasks are suitable operationalizations of luck, effort, and talent would strengthen the methodological rationale.

We have included a short paragraph (lines 118-123) to better justify our choice of the tasks, pointing to the analysis in Luhan, Poulsen & Roos (2019) who showed that the income from these tasks are in fact perceived to rely on luck, effort, and talent.

In sum, this is a well-executed and thought-provoking paper that makes a valuable methodological and conceptual contribution. With a minor clarification of the task rationale and a few stylistic improvements, it will be fully ready for publication.

We thank you for the positive assessment of our work. We have revised the manuscript to eliminate any remaining grammatical and stylistic issues that had escaped our attention previously.

Reviewer #2: Reviewer report

1. Overall assessment and major critique

The manuscript addresses a potentially important methodological question regarding how monetary framing influences fairness evaluations in economic experiments. While the topic is relevant and the experimental approach shows some thoughtful elements, a fundamental methodological confound severely undermines the validity of the paper's central conclusions.

The authors claim to demonstrate that "knowledge of monetary value can serve as a frame" that intensifies self-serving bias in fairness evaluations. However, the experimental design fails to isolate the monetary framing effect from another critical variable: the transition from evaluating multiple hypothetical scenarios to assessing a single, concrete, payoff-relevant outcome. This confound represents a fatal flaw in the study's internal validity.

We thank the reviewer for the careful reading of our manuscript and for engaging critically with the methodological aspects of our design. We appreciate the concern raised about a potential confound and have examined it closely. As outlined in our responses below, we believe that our experimental structure does allow us to draw the conclusions we present, but we agree that a more explicit acknowledgment of this potential limitation increases the transparency and value of the paper. Below, we explain our reasoning in more detail, describe the changes we have made, and hope that the expanded discussion provides a transparent and balanced account of why this does not compromise the core conclusions.

2. Detailed methodological critique

2.1 The Critical Confound: Bundled Treatments

The experimental procedure simultaneously introduces two distinct psychological treatments:

• TREATMENT A (Monetary Framing): Conversion of abstract tokens to concrete monetary values (4¢, 2¢, 3¢)

• TREATMENT B (Outcome Concretization): Identification of one specific pair as the actual determinant of final payment

The current design administers both treatments concurrently before the final evaluation. The observed shift in fairness ratings could therefore be attributed to:

• Treatment A alone (as the authors claim)

• Treatment B alone (the act of making an outcome real)
• An interaction between A and B
The literature strongly suggests that Treatment B—moving from hypothetical to real stakes—is itself a powerful driver of self-interested evaluation. The personal salience of knowing "this specific outcome determines my payment" likely triggers emotional engagement and self-serving bias independent of monetary quantification.

Thank you for the detailed explanation of your concern. In the reasoning above, two distinct issues appear intertwined:
(1) a possible transition from hypothetical- to real-stakes decisions, and
(2) the fact that one of the six evaluated distributions is selected for payment (“outcome concretization”) which the reviewer interprets as hypothetical distributions.
After carefully considering both points, we believe that neither interpretation threatens the internal validity of our design or the conclusions we draw. We address them in turn.

1) Hypothetical vs. real stakes:
We would like to clarify that participants do not move from hypothetical to real stakes between the two evaluations. If such a transition occurred, a large literature indeed shows that behavior often becomes more self-interested when decisions acquire real monetary consequences (e.g., Camerer & Hogarth 1999; Bühren & Kundt 2015; Konow 2004). For the sake of completeness, we also have to state that there are studies that report no difference in fairness or social values between hypothetical vs. real payoffs (e.g., Ben-Ner et al. 2008; Mentzakis, & Mestelman 2013). While, like the reviewer, we rather follow the former literature, we have to argue that the methodological critique does not apply to our study. In our case both fairness ***evaluations*** *are not payoff relevant, so could be interpreted as hypothetical: evaluating a distribution—either in tokens or in its monetary equivalent—has no effect on participants’ payoffs, and the allocation itself is never changed due to their evaluation. Participants therefore remain in a “non-consequential” domain throughout. The observed shift in fairness evaluations cannot therefore be attributed to incentive salience or payoff relevance but instead provides evidence that monetary framing affects fairness judgments even when consequences do not change.*

In response to your comment, we carefully reviewed the manuscript to ensure that the hypothetical nature of both decisions is clearly introduced. We added clarifications on p. 1, lines 20-21, when the setting is first described, and on p. 4, lines 143-144 abd 149-150, in addition to the existing statement on lines 138-139.

2) Selecting one pair for payment:
It is correct that we first displayed the payoffs of all six pairings, asking for fairness and happiness ratings. Participants were clearly instructed that any of the presented distributions could be selected for payoff: “At the end of the experiment,one of these six rounds will be selected and you will be paid according to the points you have after the distribution in this round.” (p. 7 in the instructions that can be found on OSF https://osf.io/jucev/). This does not mean, however, that before revealing the payoff-relevant distribution, these would be seen as hypothetical. Selecting one of several decisions for payment is a theoretically validated and commonly accepted

method in experimental economics. Random-payment mechanisms (Holt 1986; Starmer & Sugden 1991; Charness, Gneezy & Halladay 2016; Azrieli, Chambers & Healy 2018,) preserve incentive compatibility and prevent cross-task hedging or wealth effects, ensuring that each decision is evaluated independently. Empirical studies (Beattie & Loomes 1997; Cubitt, Starmer & Sugden 1998; Hey & Lee 2005) also show that paying only one decision rather than all does not systematically alter behavior. This method is widely used in fairness and distributional-preference experiments (e.g., Frohlich & Oppenheimer 1992; Blanco et al. 2011, Clott et al. 2018; Baltussen et a.l 2025). For these reasons, selecting one distribution out of six as the payoff-relevant one is not expected to bias participants' fairness judgments in our setting.

To be transparent and forthcoming with the reader regarding your second concern, we elaborate on this point in the conclusion as follows: "Given our experimental design, participants were given two points of information before making their final evaluation: the value of the tokens and the pair that was selected for payment. We base our conclusions on two established theories, self-interest [40] and motivated reasoning (self-serving bias) [10]. One could argue that learning the payoff-relevant pair might also affect the evaluation, but to the best of our knowledge, there is no theoretical background that would support this argument. The literature that appears the closest considers paying one experimental decision vs. paying all, and Charness et al. [18] found that paying one out of several periods does not alter behavior significantly." (p. 7, lines 254-261)

2.2 Required Design Modification

To properly test the authors' hypothesis, the experimental procedure should have isolated the monetary framing effect. A methodologically sound approach would be:

1. Reveal the payoff-relevant pair immediately after the earning phase
2. Collect initial fairness evaluations for this known payoff-relevant pair (in tokens)
3. Reveal monetary values and collect final evaluations

This design would complete the abstract-to-concrete transition before the first measurement, allowing any subsequent rating changes to be more confidently attributed to the monetary frame itself.

Thank you for this straightforward proposal for an improved design. We hope that our extended explanation above was convincing that this is not necessary.

P 3. Implications for interpretation

Given this confound, the authors cannot validly claim that "knowledge of monetary value" causes the observed shift in fairness evaluations. The alternative explanation—that outcome concretization drives the effect—is equally plausible based on the presented data.

The paper's central contribution ("demonstrating that knowledge of monetary value can serve as a frame") therefore remains unsubstantiated. The results may instead demonstrate that making outcomes real (rather than specifically monetary) triggers self-serving evaluations.

As stated above, we discuss the potential influence of two factors in the conclusion. We hope that our arguments above demonstrate that the reveal of the payoff relevant distribution is unlikely to drive our observations, and that we cannot see a theoretical argument for this. Luhan et al. 2019 report a comparison between fairness preferences in hypothetical and incentivized situations, using a similar production setting. They report that the self-serving bias in these situations rather leads to a switch in fairness principles than a re-evaluation of fairness of distributions.

4. Additional editorial notes

The manuscript contains several minor grammatical and phrasing issues that require attention, including inconsistent hyphenation in "crowding-out," missing possessive apostrophes in references to theoretical concepts, subject-verb agreement errors, and inconsistent use of terminology between "satisfaction" and "happiness." While these do not affect the scientific argument, they detract from the manuscript's polish.

Thank you for drawing our attention to this. We agree that the presentation of the paper is important for communicating our findings effectively. We have revised the manuscript to eliminate the grammatical issues you identified. We would also like to thank you for noticing the inconsistent use of "satisfaction" and "happiness," as this could have caused confusion.

5. Final recommendation

The identified methodological confound is fundamental to the experimental design and cannot be addressed through reanalysis or revision. The study fails to provide compelling evidence for its central thesis due to this validity threat. I therefore recommend rejection.

The research question remains valuable, and I would encourage the authors to address this design issue in future work exploring the relationship between monetary framing and fairness evaluations.

Thank you for your thoughtful engagement with the manuscript and for recognizing the broader importance of the question we examine. We hope that the clarifications provided above convincingly explain why our design is suitable for addressing it, and that the changes we have made to the text make these points also clear to the reader.

Best regards

Wolfgang Luhan

(for the authors)
